# Prompt recognition of stump appendicitis is important to avoid serious complications: a case report

**DOI:** 10.4076/1757-1626-2-7415

**Published:** 2009-07-09

**Authors:** Ismail Ismail, Domenico Iusco, Marcello Jannaci, Giuseppe Giovanni Navarra, Antonio Grassi, Serena Bonomi, Roberta Parpanesi, Andrea Giombi, Salvatore Virzì

**Affiliations:** 1 Surgical Division, AUSL Bologna, Ospedale di BentivoglioVia Marconi 35, 40010 Bentivoglio (Bologna)Italy; 2 Radiology Division, AUSL Bologna, Ospedale di BentivoglioVia Marconi 35, 40010 Bentivoglio (Bologna)Italy

## Abstract

**Introduction:**

Stump appendicitis is a rare complication of appendectomy due to recurrent inflammation of the residual appendix. The diagnosis is often delayed due to low index of suspicious, which may result in serious complications.

**Case presentation:**

We describe a case of stump appendicitis occurred 12 months after appendectomy in 25 years old man. Despite past medical history of appendectomy the diagnosis was made by means of ultrasound scan and an high degree of clinical suspicion.

**Conclusions:**

Stump appendicitis is a rare but important complication of appendectomy, often misdiagnosed. Prompt recognition is important to avoid serious complications. This pathologic entity should always be kept in mind on case of right lower quadrant pain.

## Introduction

Stump appendicitis (SA) is a rare complication of appendectomy caused by infection of the residual portion of the appendix left in place. The clinical presentation of SA does not differ from that of acute appendicitis. Although unusual, it must be included in the differential diagnosis of right lower quadrant pain in patients who already underwent appendectomy [1]. Recognition of this entity is important because delayed diagnosis may cause serious complications. A few number of stump appendicitis are reported in the medical literature [2]. We report a case of stump appendicitis in a 25-year old man, who underwent an open appendectomy one year before the admission to our Institution.

## Case presentation

A 25-year-old Caucasian man was admitted with a 24-hr history of central abdominal pain, which radiated to the right lower quadrant. The pain worsened when the patient moved, strained and coughed. He did not complain any vomiting, urinary tract symptoms or change in bowel habits. His past medical history showed an open appendectomy operation performed in another hospital one year before the admission to our Institution.

His axillary temperature was 37.3°C, rectal temperature 38.2°C. Base line investigations showed white blood cells 12.030/mmc with slight neutrophilia at 9.170/mmc and C-reactive protein 4.4 mg/dL. Urinalysis was normal.

Physical examination showed rebound tenderness in right iliac fossa during abdominal palpation in the proximity of a well-healed McBurney’s’s incision scar. Plain abdominal X-Ray revealed radiopaque image of about 1.5 cm in right lower quadrant (RLQ). Abdominal ultrasonography showed a hypoechoic oval mass that contained a hyperechoic formation and a small amount of fluid in RLQ.

**Figure 1.**
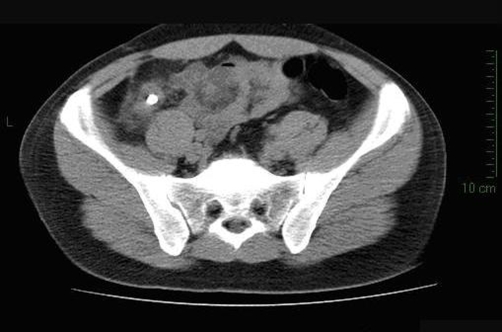
Computed Tomography scan showing a hyperdense formation in RLQ surrounded by an edematous and spastic viscera.

In the same day a computed tomography was performed, which confirmed the existence of a hyperdense formation in RLQ surrounded by an edematous and spastic viscera (Figure 1). The patient was treated with i.v. fluids and antibiotics.

**Figure 2.**
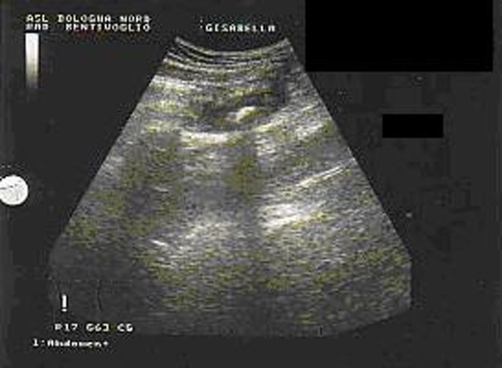
Abdominal ultrasonography showing an oval hypoechoic formation arising from the base of the cecum and the hyperechoic formation in it.

In the next day an abdominal ultrasonography was repeated revealing that, despite the past medical history of appendectomy, the oval formation arising from the base of the cecum could have been a residual appendix and the hyperechoic formation an appendicolith (Figure 2).

**Figure 3.**
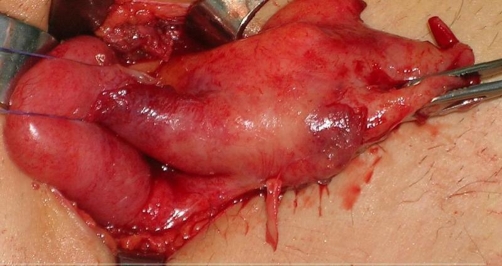
Intraoperative finding.

The patient underwent explorative laparotomy. The McBurney’s incision previously made was used, and a mass (6 × 3 × 1.5 cm) was found under the ilocaecal junction (Figure 3), which proved to be a stump appendicitis with a faecolith (1.5 cm) in it. Appendicectomy was performed, and the stump was inverted into the cecum using purse string suture, a drain was placed in the site of the operation. Post-operative course was uneventful and he was discharged 5 days after admission. The histologic evaluation reported suppurative stump appendicitis.

## Discussion

Postoperative complications after appendectomy include wound infection, intra-abdominal abscess, retrocecal abscess, intestinal perforation with peritonitis, bleeding and adhesions [3]. Stump appendicitis is one of the rare delayed complications of appendectomy first described in 2 patients by Rose in 1945 [4]. The incidence of stump appendicitis is about 1 in 50 000 cases [5] even though the real incidence is probably higher due to underestimating of this entity [6]. A modern review found none more than 37 cases reported in surgical literature. The time intervals from the initial operation ranged from 2 months to 50 years [7].

Stump appendicitis is an uncommon cause of acute abdominal pain, often mimicking other acute abdominal conditions. The most common symptoms and signs are periumbilical pain localized to the right lower quadrant, nausea, anorexia, vomiting, pyrexia, right lower quadrant tenderness, muscular guarding and rebound tenderness [8].

A correct preoperative diagnosis of stump appendicitis can be made by ultrasonography and by computed tomography. Ultrasonography can reveal a thickened appendix stump, fluid in the right iliac fossa and edema of caecum. Abdominal sonography has become the method of choice for the diagnosis of acute appendicitis since Puylaert's technique was adopted [9]. In our case sonography identified inflammatory changes present in the appendiceal stump and it was capable of revealing the existence of a faecolith inside the stump and fluid outside it.

Computed tomography (CT) findings may be similar to those present in acute appendicitis (i.e. contrast enhancing tubular structure arising from the cecum with adjacent fat strand) if the appendiceal stump left after appendectomy is long [10]. CT may also demonstrate a pericecal phlegmon or abscess, as well as a thickening of the cecal wall with oral contrast material insinuating into the expected location of appendiceal origin, the so called “arrowhead sign” [11]. In our case a tubular structure with a faecolith in it was identified but was interpreted, due to the anamnesis of previous appendectomy, as a stenotic intestinal loop with a faecolith inside it.

We think in agreement with other authors, that in most cases the diagnosis of stump appendicitis may be made by ultrasonography alone. An high index of suspicion and a certain familiarity with sonographic finding are necessary and sufficient prerequisites for an early diagnosis, without the use of more sophisticated examinations such as CT, MRI, barium enema or colonoscopy [12].

Laparoscopy has an important role in the diagnosis of stump appendicitis, this diagnostic modality may also be therapeutic [6,13]. The recognition and the adequate treatment of this acute condition is important since residual appendix may cause small bowel obstruction [14], hemorrhage from the mesoappendix [5], generalized peritonitis [15], retrocecal abscess [16]. Rarely, moreover, malignancy [17] and endometriosis may originate in the appendiceal stump [18].

The causes of stump appendicitis are: insufficient inversion of the stump, long proximal remnant of the appendix, incomplete removal of the distal remnant and partial laparoscopic or laparotomic appendectomy [3,5,19].

There are three basic methods for treating the appendiceal stump: 1) simple ligation, 2) ligation and inversion, 3) inversion without ligation. No agreement exists on which is the best method: in fact while the famous 20th century surgeon William Mayo, in explaining his preference for the simple ligation of the stump stated: “I have not had occasion to regret non having inverted the stump”. Rao et al [20] showed that all cases reported in literature undergo simple ligation of the appendix without invagination of the stump, suggesting that simple ligation with failure to amputate the appendix close to its origin from the cecum is a prerequisite for developing stump appendicitis. On the other hand, Mangi and Berger [5] reviewed 2,185 case of appendectomy and found no correlation between simple ligation and stump appendicitis. They reported that the stump must be shorter than 3 mm in depth, while other authors reported that leaving an appendix stump less than 5 mm can minimize the incidence of stump appendicitis. Infact a stump longer than 5 mm may become a reservoir for a fecolith that can perforate the stump itself [15].

It has been reported that the increasing prevalence of this rare complication may be due to the rapid development of laparoscopic appendectomy that prompted the recognition of stump appendicitis as an entity.[13] Recent reports have pointed out that the laparoscopic techniques itself may play a role in the increased incidence. Infact Uludag et al [2] suggested that the potential limitation of laparoscopy such as smaller field of vision, lack of three-dimensional perspective, absence of tactile feedback may increase the chance to leave a longer stump which may result in chronic inflammation.

Even though the first operation was not performed laparoscopically we assume that the intraoperative finding of intense inflammation may have misled the operating surgeon to leave a very long stump. The appendix in fact arises from the posterior -medial wall of the cecum about 3 cm below the ileocecal valve. Its variable position in relation to the cecum and terminal ileum, combined with acute inflammation, may result in misidentification of the appendix-caecum junction. This may lead to incomplete removal of appendix. In particular, appendectomy can sometimes be carried out without complete dissection in retrocecal subserous appendicitis. Consequently, to avoid stump appendicitis, the appendix must be dissected carefully from the top to the base before resection, and the identification of the appendiceal-cecal junction seems to play a pivotal role to avoid residual appendix. For the identification of the appendiceal-cecal junction, it is important to dissect and ligate the recurrent branch of the appendiceal artery as this mark the true base of the appendix and to follow the taenia coli of the cecum to the base [6].

## Conclusions

In conclusion, stump appendicitis is a rare but serious complication of appendectomy, often confused with other conditions. The prevalence and the incidence of stump appendicitis has been increasing in recent years, probably due to the increased use of laparoscopic approach to appendectomy. Prompt recognition is important to lead to early treatment, thus avoiding serious complications. It must be clear that the history of prior appendectomy, especially when performed laparoscopically does not rule out the possibility of a stump appendicitis. High degree of suspicion can help to make a correct diagnosis and a safe treatment. Therefore, clinicians should always keep in mind the possibility of this complication as the cause of right lower quadrant pain.

## Abbreviations

CT: Computed tomography
RLQ: Right lower quadrant
SA: Stump appendicitis
